# Gold(III) Compounds as New Family of Anticancer Drugs

**DOI:** 10.1155/S1565363303000141

**Published:** 2003

**Authors:** Luigi Messori, Giordana Marcon, Pierluigi Orioli

**Affiliations:** Department of Chemistry University of Florence via della Lastruccia 3 Florence 50019 Sesto Fiorentino Italy

## Abstract

Gold(III) complexes are emerging as a new class of metal complexes with outstanding cytotoxic properties and are presently being evaluated as potential antitumor agents. This renewed interest is the result of recent studies in which various gold(III) complexes have been shown to be stable under physiological conditions and to manifest relevant antiproliferative properties against selected human tumor cell lines. The pharmacological investigation of some representative gold(III) complexes has been extended to consider their effects on the cell cycle and to reveal induction of apoptosis. Remarkably, preliminary studies suggest that the interactions in vitro of gold(Ill) complexes with calf thymus DNA are weak whereas significant binding to model proteins takes place. Our findings imply that the mechanism of action of cytotoxic gold(Ill) complexes might be substantially different from that of clinically established platinum compounds.


### Gold(III) Compounds as New Family of Anticancer Drugs

Luigi Messori*, Giordana Marcon and Pierluigi Orioli

*Department of Chemistry, University of Florence, via della Lastruccia 3, 50019 Sesto Fiorentino (Florence), Italy.*

(Received February 21, 2003; Accepted March 14, 2003)

**ABSTRACT**

Gold(III) complexes are emerging as a new class of metal complexes with outstanding cytotoxic properties and are presently being evaluated as potential antitumor agents. This renewed interest is the result of recent studies in which various gold(III) complexes have been shown to be stable under physiological conditions and to manifest relevant antiproliferative properties against selected human tumor cell lines. The pharmacological investigation of some representative gold(III) complexes has been extended to consider their effects on the cell cycle and to reveal induction of apoptosis. Remarkably, preliminary studies suggest that the interactions in vitro of gold(III) complexes with calf thymus DNA are weak whereas significant binding to model proteins takes place. Our findings imply that the mechanism of action of cytotoxic gold(III) complexes might be substantially different from that of clinically established platinum compounds.

### GOLD(III) COMPLEXES AS A POSSIBLE ALTERNATIVE TO PLATINUM COMPLEXES.

Following the discovery of the antiproliferative effects of cisplatin, attention was devoted to gold(III) complexes as a possible alternative to antitumor platinum drugs [1-3]. This interest mainly originated from the observation that both platinum(II) and gold(III) complexes possess the same electronic configuration ($d^8$) and give rise preferentially to square planar complexes. Ligand exchange kinetics is relatively slow in both cases, although faster in gold(III) complexes. Thus, the strict relationship to platinum(II) compounds rendered gold(III) complexes attracting candidates for development and testing as potential anticancer drugs;

* Corresponding Author:
Dr. Luigi Messori
Fax: +39 055 4573385; Tel: +39 055 4573284;
e-mail: luigi.messori@unifi.it unfortunately their relatively poor chemical stability in solution heavily hindered such studies for a long time. Thus, until the mid-90's, only a few reports existed in the literature describing the cytotoxic properties and the *in vivo* antitumor effects of gold(III) complexes [4-6]. In some cases important systemic toxic effects, produced by gold(III) complexes, had been reported [1] and significant differences in the spectrum of action had been noticed compared to cisplatin.

### STRUCTURAL FEATURES OF SOME GOLD(III) COMPLEXES.

In recent years, owing to the contributions of a few research groups, new gold(III) compounds have been synthesized and characterized that show sufficient stability under physiologically relevant conditions. Such stabilization is generally achieved through an appropriate choice of the ligands, in most cases bearing nitrogen atoms as donor groups. A number of these gold(III) compounds are shown in Figure 1.

Recently some organogold(III) compounds, bearing the bipyridyl moiety, have been developed by the group of Giovanni Minghetti in Sassari (Italy); typical compounds of this family are shown in Figure 2 (the crystal structures are available for most of these gold(III) complexes).

### BEHAVIOUR IN SOLUTION OF SELECTED GOLD(III) COMPLEXES.

Essential requirements for the pharmacological evaluation *in vitro* of new metal complexes as cytotoxic agents are an appreciable solubility in water and a sufficient chemical stability under physiologically relevant conditions. Thus, prior to any biological testing, the chemical behaviour in solution of newly prepared gold(III) complexes is analysed in detail. The chemical characterization in solution is mainly performed with the help of various spectroscopic techniques including visible absorption spectroscopy, $^1$H NMR and ESI-MS [3]. Notably, most gold(III) complexes prepared in our laboratory exhibit characteristic and intense transitions in the visible that have allowed us to test their time-dependent stability. Generally, the visible spectra recorded after dissolving the individual compounds in the reference buffer (phosphate buffer, pH 7.4, NaCl 4mM or 100mM) do not show significant modifications for several hours, implying that the gold(III) chromophore does not undergo major transformations. An example is shown in Figure 3.

Subsequent application of ESI-MS and NMR techniques has allowed, in several cases, detailed identification of the species that are dominant in solution [3]. In various cases, the stability of these gold(III) complexes has been further assessed in RPMI, the culture medium used for the *in vitro* cytotoxicity experiments (unpublished results from our laboratory).

### *IN VITRO* CYTOTOXICITY AS THE MAIN SCREENING METHOD.

Screening of novel compounds for anticancer activity is a major issue for the discovery of new agents with encouraging pharmacological properties. Presently, the method adopted by NCI is based on *in vitro* tests of cytotoxic activity on a panel comprising 60 human tumor cell lines.

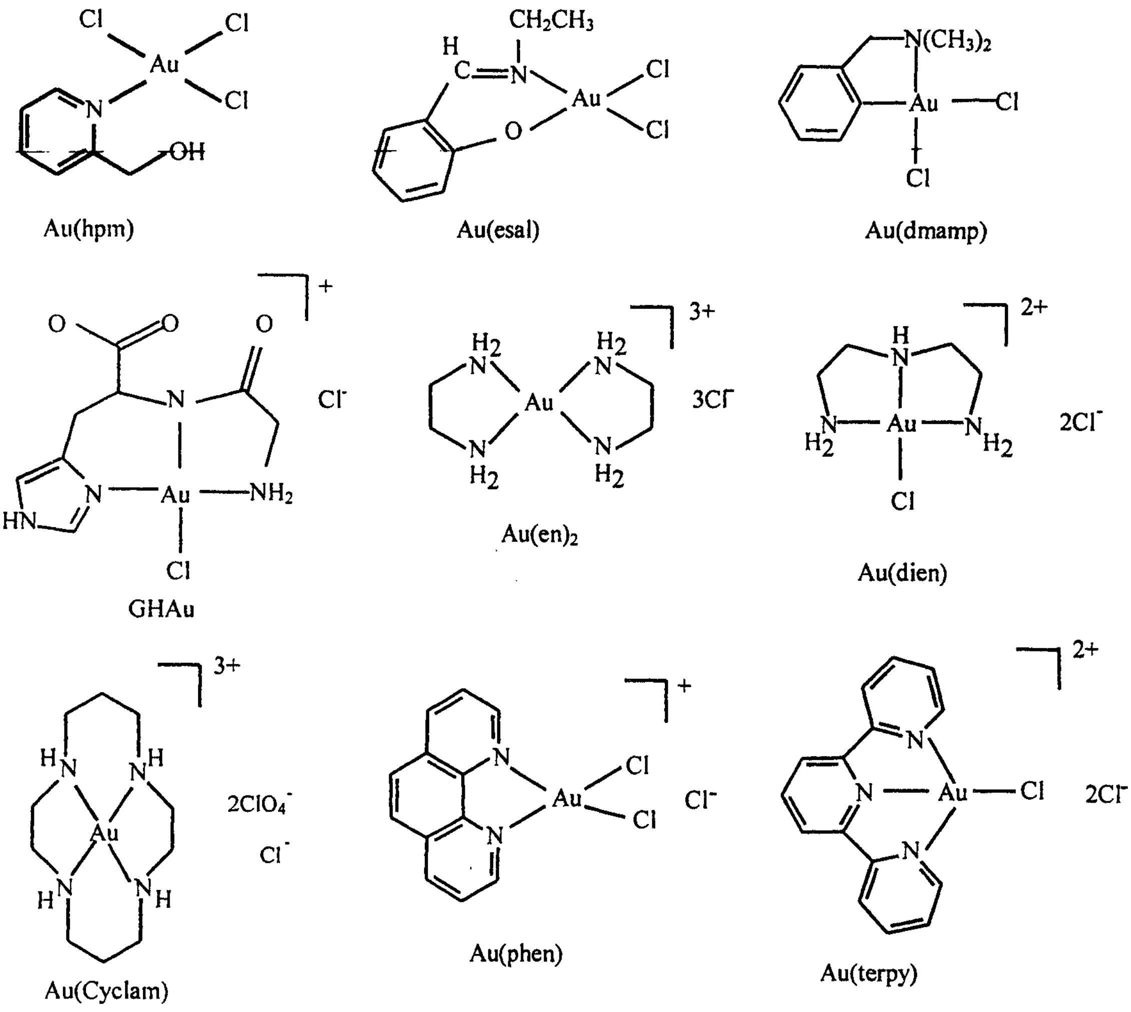

**Fig. 1:** Schematic drawing of some gold (III) complexes.

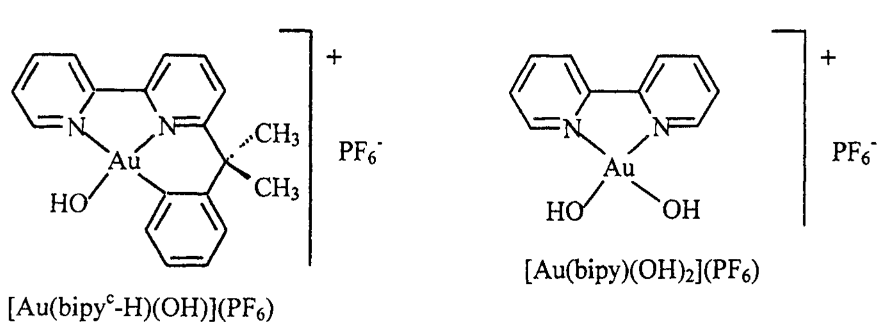

**Fig. 2:** Schematic drawing of two representative organogold(III) compounds, bearing the bipyridyl moiety.

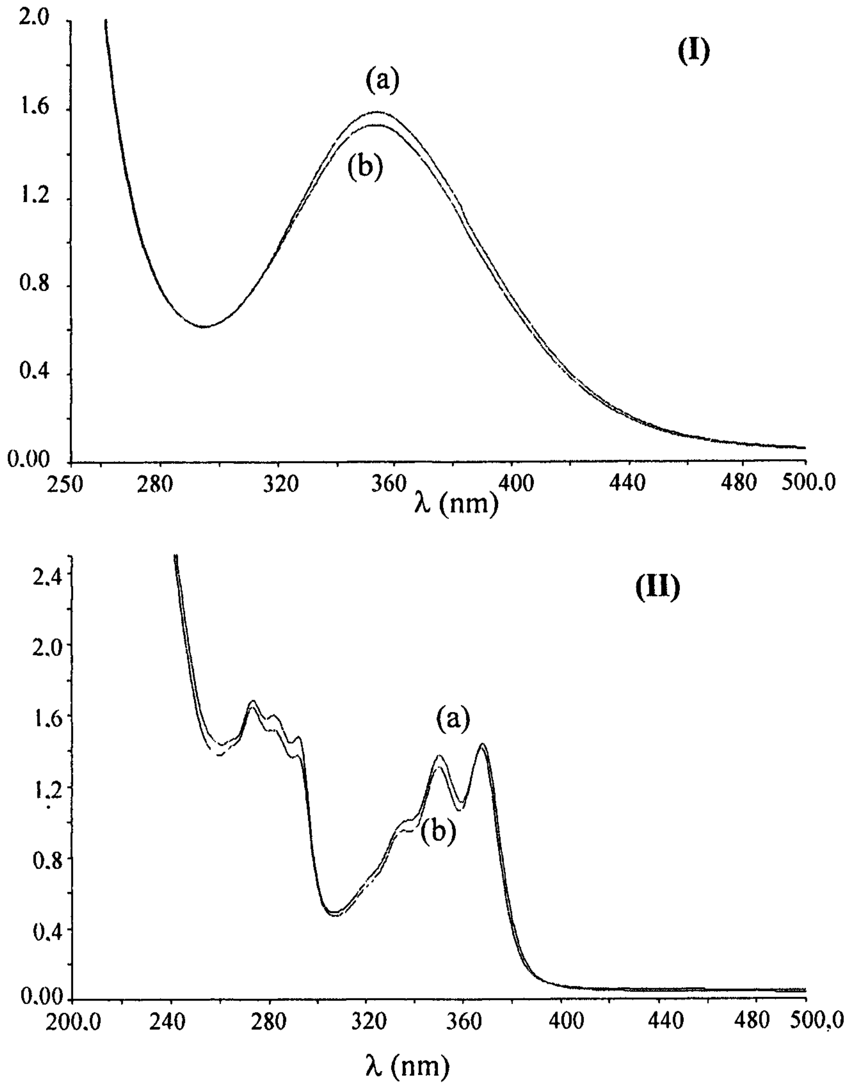

**Fig. 3:** Electronic spectra of (**I**) Au(Cyclam) $1x10^{-3}$M at mixing (a) and after 72h (b); (**II**) Au(terpy) $1,2x10^{-1}$M at mixing (a) and after 72h (b) in the reference buffer.

Thus, as a first step for their pharmacological evaluation, the activity of some novel gold(III) complexes developed in our laboratory was assayed against a small number of tumor cell lines. This restricted panel comprised two ovarian carcinoma human cell lines, A2780 (both sensitive and resistant to cisplatin) and SKOV3, the colon carcinoma HCT8 cell line and the T-lymphoblastoid leukemia CCRF-CEM cell line (both sensitive and resistant to cisplatin) [3]. Cell lines were maintained in RPMI 1640 medium supplemented with 10% horse serum (CCRF-CEM/S, CCRF-CEM/R and HCT8) or fetal bovine serum (FBS) (SKOV3, A2780/S and A2780/R) and antibiotics at 37°C in a 5% $CO_2$ atmosphere and subcultured twice weekly. Experiments were conducted on exponentially growing cells. The Sulphorhodamine-B (SRB) assay was performed in 96-well plates, using RPMI 1640 medium+ 5% FBS, according to the protocol of Skehan [9]. The concentration range used for each gold(III) complex was between $10^{-2}$ μM and 200μM. Notably, the $IC_{50}$ values recorded for several gold(III) complexes on the A2780 human tumor cell line are reported in Table 1.

**Table 1**

$ED_{50}$* Values (μM) of some gold(III) compounds in human ovarian cells A2780, both sensitive and resistant to cisplatin, after 72 hours of exposure to the drugs.

| **$ED_{50}$* (μM)** | | | | | |
|---|---|---|---|---|---|
| Complex | **A2780/S** | exp.n. | **A2780/R** | exp. n. | R/S |
| Au(hpm) | 10.1±1.0 | 3 | 21.0 | 2 | 2.1 |
| Au(esal) | 2.1±0.7 | 4 | 3.8±1.4 | 4 | 1.8 |
| GHAu | 5.2±1.6 | 3 | 8.5±2.3 | 3 | 1.6 |
| $Au(en)_2$ | 8.4±0.8 | 3 | 17.0±4.2 | 3 | 2.0 |
| Au(dien) | 8.2±0.9 | 3 | 18.7±2.2 | 3 | 2.3 |
| Au(Cyclam) | 99.0 | 2 | >120.0 | 3 | - |
| Au(terpy ) | 0.2 | 1 | 0.4±0.03 | 3 | 1.2 |
| Au(phen) | 3.8±1.1 | 5 | 3.5±0.9 | 5 | 0.9 |
| cisplatin | 1.6±1.3 | 6 | 16.1±8.7 | 6 | 10.0 |

*Expressed as mean ± SE of at least three determinations or mean of two determinations.
[a]Ratio between $ED_{50}$ values on A2780/R cells and on the parental cell line.

It emerges that most gold(III) complexes are highly cytotoxic. Indeed, all investigated gold(III) complexes -except Au(cyclam)- exhibited important cell killing properties toward the reference A2780/S line, with $ED_{50}$ values falling in the micromolar range (from 0.3 to 10 μM). In some cases resistance to cisplatin is clearly overcome.

**Table 2**

$ED_{50}$ (μM) values of cisplatin, Au(phen) and Au(dien) in the human leukemic cells CCRF-CEM, sensitive or resistant to cisplatin. Data were collected 72 hrs after removing the drugs.

| **$ED_{50}$* (μM)** | | | | | |
|---|---|---|---|---|---|
| **Complex** | CCRF-CEM | | | | |
| | 1h | 2hrs | 4hrs | 24hrs | 72hrs≠ |
| Cisplatin | 25.9±4.2 | 9.1 | 5.3±1.2 | 0.9±0.4 | 0.7±0.1 |
| Au(phen) | 169.2±7.1 | 128.7 | 98.6 | 4.4 | 2.3 |
| Au(dien) | >600 | 500 | 259±36.7 | 58.2 | 12.6 |

*Expressed as mean ± SE of at least three determinations or mean of two determinations. ( ): Ratio between $ED_{50}$ values on CCRF-CEM/CDDP cells and on the parental cell line. ≠ Data were collected immediately after 72 hrs exposure to drugs.

**Table 3**

$ED_{50}$ (μM) values of cisplatin, Au(phen) and Au(dien) in the human leukemic cells CCRF-CEM, resistant to cisplatin. Data were collected 72 hrs after removing the drugs.

| $ED_{50}$* (μM) | | | | | |
|---|---|---|---|---|---|
| **Complex** | CCRF-CEM/R | | | | |
| | 1h | 2hrs | 4hrs | 24hrs | 72hrs≠ |
| Cisplatin | >186 (>7) | 119.8 (13.2) | 66.2 (12.5) | 29.4±1.1 (34) | 19.8 (28.3) |
| Au(phen) | 214 (1.3) | 111.9 (0.9) | 90 (0.9) | 7.4 (1.7) | 6 (2.6) |
| Au(dien) | >600 | 590 (1.2) | 324 (1.3) | 163.3±39 (2.8) | 32.7±6.6 (2.6) |

*Expressed as mean ± SE of at least three determinations or mean of two determinations. ( ): Ratio between $ED_{50}$ values on CCRF-CEM/CDDP cells and on the parental cell line. ≠ Data were collected immediately after 72 hrs exposure to drugs.

In the CCRF-CEM leukemic cell line (Tables 2 and 3 ) we observed, at least qualitatively, that the longer the exposure time, the lower is the drug concentration required to kill cells. In some cases, however, the relationship between the cytotoxic potency and the exposure time is not linear, owing to summation of various contributions.

[Au(phen)$Cl_2$]Cl exhibits a profile of cytotoxicity similar to that of cisplatin on the sensitive line, although the actual concentrations that are needed to achieve the same effects are three times higher. Remarkably, [Au(phen)$Cl_2$]Cl largely overcomes resistance to cisplatin, so that it is at least three times more effective than cisplatin itself on the resistant line.

[Au(dien)Cl]$Cl_2$ is much less effective than [Au(phen)$Cl_2$]Cl, on both cell lines, although their time-dependent profiles of cytotoxicity are qualitatively similar.

Both bipyridyl gold(III) complexes were shown to produce important cell killing effects on A2780, SKOV3 and CCRF-CEM lines (Table 4). The $IC_{50}$ values of 8.8 μM and 3.3 μM for A2780 match those of other gold(III) complexes, determined under identical conditions. In particular [Au(bipy$^c$-H)(OH)][$PF_6$] qualifies as one of the most active gold(III) complexes on the A2780 line. Lower but still important cytotoxic effects were detected when both complexes were tested on the SKOV3 and CCRF-CEM cell lines. Remarkably both compounds are able to overcome to a large extent resistance to cisplatin as witnessed by the relatively low resistance index values.

**Table 4**

Inhibitory effects of gold(III) complexes on the growth of some cisplatin-sensitive (A2780/S, CCRF-CEM/S) and -resistant (A2780/R, SKOV3, CCRF-CEM/R) human tumor cell lines.

| **$ED_{50}$* (μM)** | | | |
|---|---|---|---|
| Cell lines | $[Au(bipy)(OH)_2][PF_6]$ | $[Au(bipy^c\text{-}H)(OH)][PF_6]$ | Cisplatin |
| A2780/S | 8.8±3.9<br>n=4 | 3.3±1.4<br>n=5 | 1.3±0.2<br>n=5 |
| A2780/R | 24.1±8.7<br>n=6<br>(2.7) | 8.2±1.5<br>n=7<br>(2.5) | 15.3±1.9<br>n=5<br>(11.7) |
| SKOV3 | 34.4±4.7<br>n=5 | 13.3±1.6<br>n=5 | 21.6±4.1<br>n=5 |
| CCRF-CEM/S | 52.9±11.6<br>n=5 | 11.9±2.1<br>n=5 | 1.0±0.3<br>n=5 |
| CCRF-CEM/R | 58.6±0.9<br>n=2<br>(1.1) | 51.2±5.6<br>n=3<br>(4.3) | 14.1±8.2<br>n=2<br>(14.1) |

*$ED_{50}$ is defined as the concentration of drug required to inhibit cell growth by 50% compared to control; ( ) Ratio of $ED_{50}$ of cisplatin-resistant cell line and $ED_{50}$ of parental sensitive cell line; n = number of determinations.

Analysis of the cytotoxicity data permits formulation of some preliminary structure/function relationships that are summarized below:

i) the cytotoxicity of these gold(III) complexes is associated to the presence of the gold(III) center (indeed $Au(en)_2$ and Au(dien) are significantly more cytotoxic than the corresponding platinum compounds); ii) the presence of hydrolysable chloride groups on the gold(III) center or, more in general, of good leaving groups, does not represent an essential requirement for cytotoxicity; iii) excessive stabilization of the gold(III) center results in loss of biological activity (Au(cyclam), with the gold(III) center tightly bound to the macrocycle cage, is not cytotoxic, probably as a consequence of its low reactivity).

iv) The amount of gold that enters cells is roughly proportional to the exposure time, at least during the first hours. Thus, at least qualitatively, the longer the exposure time, the lower the drug concentration required to kill cells.

Remarkably, most of the gold(III) compounds are able to overcome to a large extent resistance to cisplatin as witnessed by the relatively low resistance index values, suggesting a different mechanism of action with respect to cisplatin.

### MORE ADVANCED BIOLOGICAL STUDIES ON CELLS: THE CELLULAR EFFECTS OF TWO REPRESENTATIVE GOLD(III) COMPLEXES.

The cell growth inhibition assay represents the standard criterion for the screening of antitumor compounds. However, this approach does not give direct information on the mechanism of action of the individual drugs. To better elucidate the possible mechanisms through which cytotoxic gold(III) complexes produce their biological effects, additional work has been carried out in our laboratory on selected tumor cell lines. Experiments were carried out on the leukemic cell population CCRF-CEM, either sensitive or resistant to cisplatin, following treatment with two representative gold(III) complexes. Specifically, the effects of Au(phen) and Au(dien) on the above cell lines were monitored by the COMET assay [10,11], and by flow cytometry studies [12]. This approach has resulted in a detailed description of cell responses to these gold(III) complexes [13].

The single-cell gel electrophoresis assay (COMET assay) is an established method to reveal DNA damage produced by drugs at the single-cell level [11,12]. The electrophoretic mobility of alkaline denatured cellular DNA is retarded by crosslinks that are quantitated as the decrease in comet tail moment, compared to $H_2O_2$-treated controls. No clear relationship can be established between the DNA damage measured by COMET and the cytotoxic activity. A possible explanation for this lack of correlation is that the cytotoxicity measured at a given time after drug exposure largely depends on the activity and the efficiency of DNA repair (excision and polymerization phenomena, for example). However, apart from difficulties in explaining some features of our experimental results, there is no doubt that the comet profiles are drastically modified by both gold(III) complexes, thus providing unambiguous evidence for a direct interaction of these gold(III) complexes with DNA.

The flow cytometry results show that both gold(III) complexes modify the cell cycle less markedly than equitoxic amounts of cisplatin. The effects induced by gold(III) complexes on the sensitive line are modest, while transient but significant modifications are observed in the resistant line. In spite of the modest effects produced on the cell cycle, both tested gold(III) complexes, but especially Au(phen), were shown to induce significant DNA fragmentation, suggestive of induction of apoptosis.

### DNA AS A POSSIBLE TARGET FOR GOLD(III) COMPLEXES: A BIOINORGANIC APPROACH

DNA is the primary target for platinum(II) antitumor compounds and is the probable target for several other antitumor metal complexes. Thus, we worked on the hypothesis that the cytotoxic effects produced by this group of gold(III) complexes on selected tumor cell lines might be a consequence of direct DNA damage; some support for this view is offered by the above described COMET experiments.

The reactions of five gold(III) complexes – $Au(en)_2$, Au(dien), Au(cyclam), Au(phen) and Au(terpy) – with calf thymus DNA were analyzed *in vitro* through a number of classical physico-chemical methods including spectrophotometry, circular dichroism, analysis of DNA thermal denaturation profiles and dialysis experiments [14]. Such investigations aimed at describing the gold(III)/DNA reaction in terms of DNA structural modifications, strength and kinetic of the interaction, nature and reversibility of gold(III)/DNA binding.

We demonstrated that the interactions of these gold(III) complexes with calf thymus DNA are relatively weak, reversible and, in most cases, electrostatic in nature. Given its chemical structure Au(cyclam) provides the best example for a "pure" electrostatic interaction. The same holds for $Au(en)_2$ that probably acts as a groove binder by analogy with the isostructural $Pt(en)_2$ complex; Au(dien) can form both electrostatic and covalent bonds. For both Au(phen) and Au(terpy) intercalative binding is possible as well as formation of coordinative bonds to nucleobases. In all cases, binding is completely reversible and gold(III) complexes are displaced from DNA by repeated ultrafiltrations.

The effects on DNA conformation produced by the individual gold(III) complexes were best detected by circular dichroism (a representative experiment is shown in Figure 4).

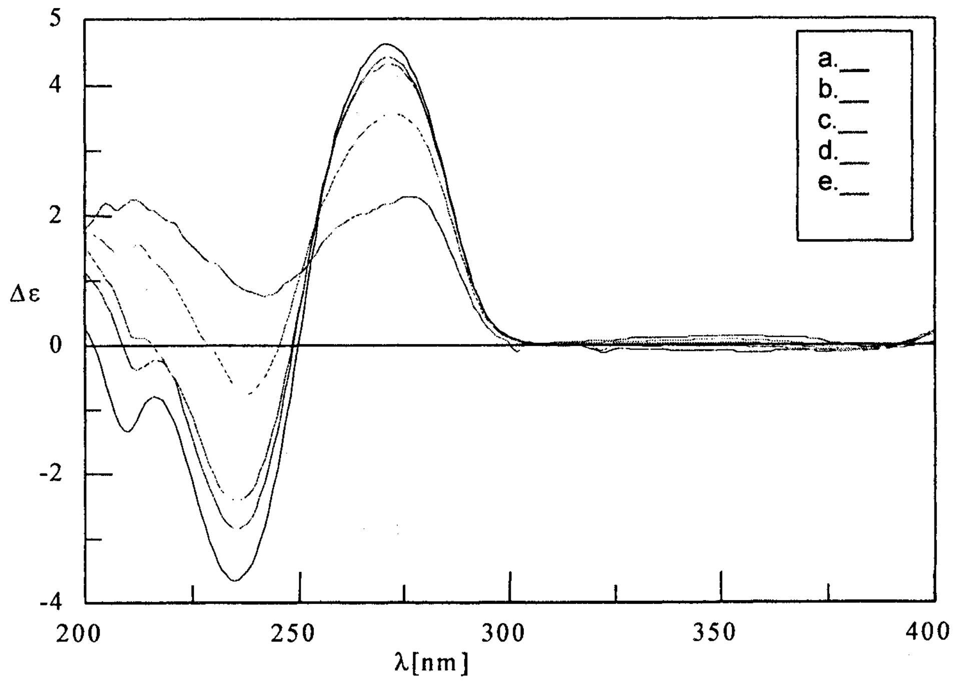

**Fig. 4:** CD spectra of calf thymus DNA (a) after addition of Au(terpy) at the ratio r = 0.1 (b), 0.2 (c), 0.5 (d), 1 (e).

$Au(en)_2$ and Au(dien) at low concentrations were shown to induce few spectral modifications, while larger effects were observed at higher concentrations still within the frame of a B-type DNA conformation. Au(cyclam) is more effective than the other polyamine complexes in inducing conformational modifications. The CD perturbations induced by Au(phen) and Au(terpy) are larger in agreement with an intercalative binding model. On the basis of the spectrophotometric results mixed binding of Au(terpy) to DNA-first intercalation, then coordination- may be hypothesized

Also the interactions of the bipyridyl compounds $[Au(bipy)(OH)_2][PF_6]$ and $[Au(bipy^c\text{-}H)(OH)][PF_6]$ with DNA were analyzed in detail through the same techniques [6]. It was found that the interactions with the DNA double helix are weak, reversible and predominantly electrostatic in nature, suggesting that DNA is not the primary target for the cytotoxic effects of these complexes.

### CONCLUSIONS

The appreciable stability of these novel gold(III) complexes under physiological conditions has allowed extensive *in vitro* pharmacological testing. These compounds have been primarily characterized through *in vitro* cytotoxictiy assays in line with the general screening strategy of NCI. Some preliminary correlations between cytotoxicity and the chemical structure have been proposed. In view of the relevant cytotoxic effects exerted by most of the investigated complexes, more detailed studies have been performed to describe cellular responses to these substance. It is worth reminding that preliminary COMET results obtained in our laboratory on Au(dien) and Au(phen) suggest that both complexes are capable of producing a direct DNA damage. Occurrence of a direct interaction of the present gold(III) complexes with DNA is also supported by CD and DNA melting experiments on *calf thymus* DNA. However, the interactions of gold(III) complexes with the DNA double helix are generally weak, reversible and predominantly electrostatic in nature, suggesting that DNA is not the primary target for the cytotoxic effects of these complexes.

### ACKNOWLEDGMENTS

This work was supported by a grant from "Ministero dell'Istruzione, dell'Università e della Ricerca" (Programmi di Ricerca Cofinanziati 2001). We thank the group of Prof. Mini (Laboratory of Pharmacology, University of Florence) for the support in carrying out cytotoxicity experiments.

### REFERENCES

1. B. Bruni, A. Guerri, G. Marcon, L. Messori and P. Orioli, *Croatica Chemica Acta*, **72**, 221-229 (1999).
2. S. Carotti, G. Marcon, M. Marussich, T. Mazzei, L. Messori, E. Mini and P. Orioli, *Chem. Biol. Interact.*, **125**, 29-38 (2000).
3. L. Messori, F. Abbate, G. Marcon, P. Orioli, M. Fontani, E. Mini, T. Mazzei, S. Carotti, T. O'Connell and P. Zanello, *J. Med. Chem.*, **43**, 3541-3548 (2000).
4. F. Abbate, P. Orioli, B. Bruni, G. Marcon and L. Messori, *Inorg. Chim. Acta*, **311**, 1-5 (2000).
5. G. Marcon, T. O'Connell, P. Orioli and L. Messori, *Metal-Based Drugs*, **7**, 253-256 (2000).
6. G. Marcon, S. Carotti, M. Coronnello, L. Messori, E. Mini, P. Orioli, T. Mazzei, M.A. Cinellu, and G. Minghetti, *J. Med. Chem.*, **45**, 1672-1677 (2002).
7. L. Messori, F. Abbate, P. Orioli, C. Tempi and G. Marcon, *Chem. Comm.*, **6**, 612-613 (2002).
8. G. Marcon, L. Messori and P. Orioli, *Expert Rev. Anticancer* Therapy, **2**, 235-244 (2002).
9. P. Skehan, R. Storeng, D. Scudiero, A. Monks, J. Mcmahon, D. Vistica, J.T. Warren, H. Bokesch, S. Kenney and M.R. Boyd, *J. Natl. Cancer Inst.*, **82**, 1107-1112 (1990).
10. R.R. Ce, The single cell gel/comet assay: a microgel electrophoretic technique for the detection of DNA damage and repair in individual cells. D.H. Phillips and S. Venit (Eds.), *Environmental Mutagenesis*, βIOS Scientific Publishers, Oxford, UK, 315-339 (1995).

11. L. Szmigiero and K. Studzian, *Anal. Biochem.*, **168**, 88-93 (1988).
12. A. Vaisman, M. Varchenko, I. Said and S.G. Chaney, *Cytometry*, **27**, 54-64 (1997).
13. M. Coronnello, G. Marcon, S. Carotti, B. Caciagli, T. Mazzei, E. Mini, P. Orioli and L. Messori, *Oncol. Res.*, **12**, 361-370 (2001).
14. L. Messori, G. Marcon, C. Tempi and P. Orioli, *Biochem. Biophys. Res. Commun.*, **281**, 352-360 (2001).

